# Acacetin Prevents Bone Loss by Disrupting Osteoclast Formation and Promoting Type H Vessel Formation in Ovariectomy-Induced Osteoporosis

**DOI:** 10.3389/fcell.2022.796227

**Published:** 2022-04-19

**Authors:** Xiao Lin, Fang Xu, Ke-Wen Zhang, Wu-Xia Qiu, Hui Zhang, Qiang Hao, Meng Li, Xiao-Ni Deng, Ye Tian, Zhi-Hao Chen, Ai-Rong Qian

**Affiliations:** ^1^ Lab for Bone Metabolism, Xi’an Key Laboratory of Special Medicine and Health Engineering, Key Lab for Space Biosciences and Biotechnology, Research Center for Special Medicine and Health Systems Engineering, NPU-UAB Joint Laboratory for Bone Metabolism, School of Life Sciences, Northwestern Polytechnical University, Xi’an, China; ^2^ State Key Laboratory of Cancer Biology, Biotechnology Center, School of Pharmacy, Fourth Military Medical University, Xi’an, China

**Keywords:** acacetin, osteoporosis, osteoclasts, type H vessel, Akt/GSK3β, NF-κB

## Abstract

Osteoporosis, characterized by the destruction of bone resorption and bone formation, is a serious disease that endangers human health. Osteoporosis prevention and treatment has become one of the important research contents in the field of medicine. Acacetin, a natural flavonoid compound, could promote osteoblast differentiation, and inhibit osteoclast formation *in vitro*. However, the mechanisms of acacetin on osteoclast differentiation and type H vessel formation, as well as the effect of preventing bone loss, remain unclear. Here, we firstly used primary bone marrow derived macrophages (BMMs), endothelial progenitor cells (EPCs), and ovariectomized (OVX) mice to explore the function of acacetin on bone remodeling and H type vessel formation. In this study, we found that acacetin inhibits osteoclast formation and bone resorption of BMMs induced by the macrophage colony stimulating factor (M-CSF) and receptor activator of nuclear factor-κB ligand (RANKL) in a concentration of 20 μM without exerting cytotoxic effects. It was accompanied by downregulation of osteoclast differentiation marker genes (*Ctsk*, *Acp5*, and *Mmp9*) and cell fusion genes (*CD9*, *CD47*, *Atp6v0d2*, *Dc-stamp*, and *Oc-stamp*). Moreover, acacetin disrupted actin ring formation and extracellular acidification in osteoclasts. Mechanistic analysis revealed that acacetin not only inhibits the expression of the major transcription factor NFATc1 and NF-κB during RANKL-induced osteoclast formation, but also suppresses RANKL-induced the phosphorylation of Akt, GSK3β, IκBα, and p65. Additionally, acacetin enhanced the ability of M-CSF and RANKL-stimulated BMMs to promote angiogenesis and migration of EPCs. We further established that, *in vivo*, acacetin increased trabecular bone mass, decreased the number of osteoclasts, and showed more type H vessels in OVX mice. These data demonstrate that acacetin prevents OVX-induced bone loss in mice through inhibition of osteoclast function and promotion of type H vessel formation *via* Akt/GSK3β and NF-κB signalling pathway, suggesting that acacetin may be a novel therapeutic agent for the treatment of osteoporosis.

## 1 Introduction

Osteoporosis is a chronic bone disease characterized by destruction of bone microstructure and low bone mass, leading to increased bone fragility and thus increased fracture susceptibility (Chujiao Lin et al., 2019). Bone resorption mediated by osteoclasts exceeds bone formation mediated by osteoblasts, which is one of the potential mechanisms of osteoporosis (Qiang Xu et al., 2021). Studies have shown that 50% of women will suffer fractures due to osteoporosis in their lifetimes (Beekman et al., 2019). Therefore, the prevention and treatment of osteoporosis has become a focus of medical and health research. In the treatment of osteoporosis, antiresorptive drugs, including bisphosphonates (alendronate, zoledronate, etc.) and denosumab, increase bone mineral density and reduce the risk of fracture by inhibiting osteoclast-mediated resorption (Leder et al., 2020). However, the cost of Western medicine therapy is high, and the clinical side effects are uncertain, such as fever, joint myalgia, hypocalcaemia, and other potential adverse events (Meng et al., 2020). Therefore, the use of traditional Chinese medicine, which has the concomitant function of both medicine and foodstuff in the treatment of osteoporosis, has the advantages of low cost and fewer side effects (Shi et al., 2020).

Osteoclasts are multinucleated cells, that differentiate from monocytes/macrophages in the presence of M-CSF and RANKL (Xiao Lin et al., 2019). When RANKL activates the receptor activator of nuclear factor-κB (RANK), the inner membrane portion of RANK reacts with tumour necrosis factor receptor-associated factor 6 (TRAF6) and activates a series of downstream signalling pathways, including AKT, nuclear factor-κB (NF-κB), mitogen-activated protein kinase (MAPK), and calcium signalling pathways (Delong Chen et al., 2020). Furthermore, the differentiation, survival, multinucleation, and activation of osteoclasts are regulated by these pathways (Dai et al., 2017). In recent years, type H vessels, a special subtype of vessel with strong expression of CD31 and EMCN in endothelial cells, have been identified to couple the balance of bone absorption and formation (Xie et al., 2014). Mononuclear preosteoclasts can promote the growth of type H vessels by releasing platelet-derived growth factor-BB (PDGF-BB) prior to multinucleated osteoclast formation (Xiaoqun Li et al., 2020). Moreover, the number of type H vessels decreases with age to a degree consistent with the severity of bone loss (Song et al., 2020). Therefore, by simultaneously interrupting the maturation and activation of osteoclasts, inhibiting bone resorption and promoting the formation of type H blood vessels, osteoporosis can be effectively prevented.

Flavonoids are abundant in many common vegetables, fruits, grains, and herbs. They have potential therapeutic properties due to their antioxidant, anti-inflammatory, differentiation, and apoptotic properties (Bellavia et al., 2021). The intake of flavonoids increases bone mineral density (BMD), reduces bone resorption in perimenopausal women, and maintains bone health (Hardcastle et al., 2011; Zhang et al., 2014). Many flavonoid compounds have been evaluated as potential alternative therapeutic candidates against bone resorptive diseases, such as cladrin, icariin, petunidin, and epiafzelechin (Bellavia et al., 2021; Yingxing Xu et al., 2021). Acacetin (5,7-dihydroxy-4-methoxyflavone) is a flavonoid compound that can be isolated from Damiana, Saussurea involucrata plant, and black locust plants (Ren et al., 2020). It exerts pronounced anti-inflammatory, anti-peroxidative, and anti-cancer activities (Wang et al., 2020). Interestingly, acacetin has been reported to promote osteoblastic differentiation and mineralization (Li et al., 2016) and inhibit osteoclastic differentiation *in vitro* (Kim et al., 2020). However, the effect of acacetin on the formation of osteoclasts and type H vessels, and its protective function in boss loss *in vivo* are still unclear.

In this study, we investigated the effects and mechanism of acacetin on RANKL-mediated osteoclastogenesis and elucidated whether this compound could attenuate osteoclast formation and promote type H vessel formation in OVX mice. Our results demonstrated that acacetin inhibits osteoclastogenesis and bone resorption through suppression of the Akt/GSK3β and NF-κB signalling pathways, as well as stimulation of type H vessels cocultured with osteoclasts, thereby preventing the OVX-induced bone loss *in vivo*.

## 2 Materials and Methods

### 2.1 Reagents and Antibodies

Acacetin (PubChem CID: 5280442) (purity >98%, HPLC) was purchased from Herbest Bio-Tech Co., Ltd. (Baoji, China). Alpha-minimal essential medium (α-MEM) was obtained from Gibco (Rockville, MD, United States). Foetal bovine serum (FBS) was obtained from HyClone (MA, United States). M-CSF and RANKL were provided by R&D (R&D Systems, MN, United States). Sodium carboxymethyl cellulose (CMC-Na), peroxidase conjugate-wheat germ agglutinin (WGA) (L3892), FITC-WGA (L4895), acid phosphatase, leukocyte (TRAP) kit, rhodamine-conjugated phalloidin, acridine orange, and dimethylsulfoxide (DMSO) were obtained from Sigma-Aldrich (St Louis, MO, United States). Antibodies against CTSK (sc-48353), ACP5 (sc-376875), MMP9 (sc-13520), NFATc1 (sc-7294), PI3K (sc-376112), p-Akt (sc-293125), Akt (sc-81434), p-GSK3β (sc-373800), GSK3β (sc-53931), p-IκBα (sc-8404), IκBα (sc-1643), p-NF-κB p65 (sc-136548), NF-κB p65 (sc-8008), p-ERK (sc-81492), ERK (sc-514302), p-JNK (sc-6254), JNK (sc-7345), p-p38 (sc-7973), p38 (sc-7972), CD31 (365804), EMCN (sc-65495), PDGF-BB (sc-365805), TRAP (sc-376875), and GAPDH (sc-166574) were obtained from Santa Cruz Biotechnology (CA, United States). ECL chemiluminescence reagents were obtained from Pierce Biotechnology (Rockford, IL, United States). The DAB Horseradish Peroxidase Colour Development Kit, One Step TUNEL Apoptosis Assay Kit, Calcein AM, 4′,6-diamidino-2-phenylindole (DAPI), paraformaldehyde (PFA), ethylenediaminetetraacetic acid (EDTA), Haematoxylin and Eosin (H&E) Staining Kit, horseradish peroxidase (HRP)-conjugated IgG, RIPA Lysis Buffer, Calcein AM, and Triton X-100 were obtained from Beyotime Biotechnology (Jiangsu, China). Matrigel was obtained from BD (Franklin Lakes, NJ, United States). TRIzol reagent and methyl thiazolyl tetrazolium (MTT) were obtained from Invitrogen (Rockville, MD, United States). A one-step PrimeScript RT reagent kit and SYBR Premix Ex TaqIIkit were provided by TaKaRa (Dalian, China). A mouse PDGF-BB ELISA kit was obtained from Jingmei Biotechnology (Jiangsu, China). A glutamic-oxalacetic transaminase (GOT/AST) activity assay kit, glutamic-pyruvic transaminase (GPT/ALT) activity assay kit, urea nitrogen assay kit, and creatinine (CREA) assay kit were obtained from Sangon Biotech (Shanghai, China).

### 2.2 Cell Culture

Primary bone marrow derived macrophages (BMMs) were isolated from the femur and tibia of six-week-old C57BL/6J mice by flushing the marrow with a syringe and then cultured in α-MEM supplemented with 10% FBS, 100 U/mL penicillin and 100 μg/ml streptomycin in the presence of 5 ng/mL M-CSF for 24 h. Then, cells in the supernatant were collected as primary BMMs and seeded for osteoclastogenic differentiation induction. Osteoclastogenesis of BMMs was induced by conditioned medium in α-MEM supplemented with 10% FBS, 100 μg/ml streptomycin and 100 U/mL penicillin in the presence of 10 ng/mL M-CSF and 10 ng/ml RANKL. The cells were seeded into 24-well plates at a density of 2 × 10^5^/well and induced with conditioned medium for 4 days in a 5% CO_2_ incubator at 37°C.

Endothelial progenitor cells (EPCs) (Newgainbio, China) were culture in endothelial growth medium-2 (EGM-2) (Lonza). Endothelial basal medium-2 (EBM-2; Lonza) was used for experiments that do not require growth factors. The cells were incubated at 37°C in a 5% CO_2_ incubator.

The MC3T3-E1 osteoblastic cell line were cultured in α-MEM supplemented with 10% FBS, 100 U/mL penicillin and 100 μg/ml streptomycin and incubated at 37°C in a 5% CO_2_ incubator.

### 2.3 Cytotoxicity Assay

BMMs were seeded into 96-well plates at a density of 4 × 10^4^/well, and cultured with α-MEM supplied with 5 ng/mL M-CSF and treated with various concentrations of acacetin (0, 1, 5, 10, 20, 50, and 100 μM). After 24 and 48 h of incubation, 0.5 mg/ml MTT was added to each well and incubated for 3 h at 37°C. Then, DMSO was used to dissolve the formazan crystals, and the optical density was measured at a wavelength of 490 nm using a microplate analyser (BioTek, United States).

### 2.4 TUNEL Staining

BMMs were seeded into 24-well plates at a density of 2 × 10^5^/well, cultured with α-MEM supplied with 5 ng/mL M-CSF and treated with various concentrations of acacetin (0, 5, 10, 20, 50, and 100 μM). After 24 and 48 h of incubation, the TUNEL staining assay was performed using a One Step TUNEL Apoptosis Assay Kit. In brief, BMMs were washed once with precooled PBS for 3–5 min. Then, the cells were fixed in 4% PFA for 30 min and washed again with precooled PBS. The cells were permeabilized with 0.3% Triton X-100 for 5 min and washed with PBS twice. After adding 50 μL TUNEL solution/well, the samples were developed in the dark for 1 h at 37°C, washed 3 times with precooled PBS and observed using an inverted fluorescence microscope (Leica, Wetzlar, Germany). The apoptosis rate of in randomly chosen areas was quantified using ImageJ software.

### 2.5 TRAP Staining

BMMs (2 × 10^5^ cells/well) were seeded into 24-well plates, incubated with 10 ng/ml RANKL and 10 ng/mL M-CSF and treated with various concentrations of acacetin (0, 5, 10, and 20 μM) for 4 days. After fixation in 4% PFA for 20 min, the cells were subjected to TRAP staining using the acid phosphatase, leukocyte (TRAP) kit according to the manufacturer’s instructions. The number of TRAP^+^ osteoclasts containing more than 3 nuclei was counted using an optical microscope (Leica, Wetzlar, Germany).

Femurs of sham, OVX and OVX + Acacetin mice were sectioned in 4-μm-thick tissue sections for TRAP staining using the same kit. The stained sections were scanned using an Aperio AT2 Digital Whole Slide Scanner (Leica, Wetzlar, Germany). The osteoclast number/endocortical surface (N.Oc/BS, 1/mm) was measured using ImageJ.

### 2.6 RNA Extraction and Real-Time PCR Analysis

BMMs were induced with conditioned medium and treated with acacetin (0, 5, 10, 20 μM) for 4 days. Total RNA was isolated from cells and tibiae using TRIzol reagent according to the manufacturer’s instructions. Reverse transcription was performed using the one-step PrimeScript RT reagent kit for mRNA analysis. Real-time PCR assays for mRNA analysis were performed using a SYBR Premix Ex TaqII kit with a Thermal Cycler C-1000 Touch System (Bio-Rad, Hercules, CA). The primers used for real-time PCR are listed in Table 1. *Gapdh* was used as an internal control for mRNA detection. All primers were purchased from Sangon Biotech (Shanghai, China).

**TABLE 1:** Primer sequences used in real-time PCR.

**Gene**	**Primer sequences**
Acp5	Forward: 5′-TCC​GTG​CTC​GGC​GAT​GGA​CCA​GA-3′
	Reverse: 5′-CTG​GAG​TGC​ACG​ATG​CCA​GCG​ACA-3′
CtsK	Forward: 5′-AGG​CAT​TGA​CTC​TGA​AGA​TGC​T-3′
	Reverse: 5′-TCC​CCA​CAG​GAA​TCT​CTC​TG-3′
Mmp9	Forward: 5′-GCG​GCC​CTC​AAA​GAT​GAA​CGG-3′
	Reverse: 5′-GCT​GAC​TAC​GAT​AAG​GAC​GGC​A-3′
CD9	Forward: 5′-CGG​TCA​AAG​GAG​GTA​G-3′
	Reverse: 5′-GGA​GCC​ATA​GTC​CAA​TA-3′
CD47	Forward: 5′-TGG​TGG​GAA​ACT​ACA​CTT​GCG​A-3′
	Reverse: 5′-AGG​CTG​ATC​CTT​GGT​CAG​TGT​TG-3′
Atp6v0d2	Forward: 5′-TCA​GAT​CTC​TTC​AAG​GCT​GTG​CTG-3′
	Reverse: 5′-GTG​CCA​AAT​GAG​TTC​AGA​GTG​ATG-3′
Dc-stamp	Forward: 5′-GGG​CAC​CAG​TAT​TTT​CCT​GA-3′
	Reverse: 5′-CAG​AAC​GGC​CAG​AAG​AAT​GA-3′
Oc-stamp	Forward: 5′-GGG​CTA​CTG​GCA​TTG​CTC​TTA​GT-3′
	Reverse: 5′-CCA​GAA​CCT​TAT​ATG​AGG​CGT​CA-3′
Atp6i	Forward: 5′-CAC​AGG​GTC​TGC​TTA​CAA​CTG-3′
	Reverse: 5′-CGT​CTA​CCA​CGA​AGC​GTC​TC-3′
c-Fos	Forward: 5′ACT​TCT​TGT​TTC​CGG​C-3′
	Reverse: 5′-AGC​TTC​AGG​GTA​GGT​G-3′
Pu.1	Forward: 5′-ACT​CCT​TCG​TGG​GCA​GCG​ATG​GAG-3′
	Reverse: 5′-GGG​AAG​CAC​ATC​CGG​GGC​ATG​TAG-3′
Nfatc1	Forward: 5′-GAG​AAT​CGA​GAT​CAC​CTC​CTA​C-3′
	Reverse: 5′-TTG​CAG​CTA​GGA​AGT​ACG​TCT​T-3′
Nf-κb p65	Forward: 5′-CAA​AGA​CAA​AGA​GGA​AGT​GCA​A-3′
	Reverse: 5′-GAT​GGA​ATG​TAA​TCC​CAC​CGT​A-3′
Rankl	Forward: 5′-GGA​AGC​GTA​CCT​ACA​GAC​TAT​C-3′
	Reverse: 5′-AAA​GTG​GAA​TTC​AGA​ATT​GCC​C-3′
Opg	Forward: 5′-ACC​AGT​GAT​GAG​TGT​GTG​TAT​T-3′
	Reverse: 5′-AGA​ATT​CGA​TCT​CCA​GGT​AAC​G-3′
Runx2	Forward: 5′-CGC​CCC​TCC​CTG​AAC​TCT-3′
	Reverse: 5′-TGC​CTG​CCT​GGG​ATC​TGT​A-3′
Alp	Forward: 5′-GTT​GCC​AAG​CTG​GGA​AGA​ACA​C-3′
	Reverse: 5′-CCC​ACC​CCG​CTA​TTC​CAA​AC-3′
Ocn	Forward: 5′-GAA​CAG​ACT​CCG​GCG​CTA-3′
	Reverse: 5′-AGG​GAG​GAT​CAA​GTC​CCG-3′
Gapdh	Forward: 5′-TGC​ACC​ACC​AAC​TGC​TTA​G-3′
	Reverse: 5′-GGA​TGC​AGG​GAT​GAT​GTT​C-3′

### 2.7 Western Blotting

Whole-cell lysates for western blotting were prepared by extracting proteins from the cells using RIPA lysis buffer, and then blotting them on a polyvinylidene fluoride membrane. The membranes were incubated with antibodies against CTSK (1:1,000), ACP5 (1:1,000), MMP9 (1:1,000), NFATc1 (1:1,000), PI3K (1:1,000), p-Akt (1:1,000), Akt (1:1,000), p-GSK3β (1:1,000), GSK3β (1:1,000), p-IκBα (1:1,000), IκBα (1:1,000), p-NF-κB p65 (1:1,000), NF-κB p65 (1:1,000), p-ERK (1:1,000), ERK (1:1,000), p-JNK (1:1,000), JNK (1:1,000), p-p38 (1:1,000), p38 (1:1,000), and GAPDH (1:5,000). Secondary antibodies conjugated with HRP (1:10,000) were used for signal detection. The blots were visualized using ECL chemiluminescence reagents.

### 2.8 Bone Resorption Assay

Bone resorption assays were performed using Osteo Assay surface (Corning, NY, United States). BMMs were seeded onto osteo surface and induced with 10 ng/mL M-CSF and 10 ng/ml RANKL for 5 days. Bone resorption pits were sonicated in PBS to remove adherent cells. The bone resorption pits on the osteo surface were visualized using optical microscope (Leica, Wetzlar, Germany).

### 2.9 Actin Staining

Primary BMMs on bone slices or glass chamber slides were induced with 10 ng/mL M-CSF and 10 ng/ml RANKL and treated with various concentrations of acacetin for 4 days. The cells were washed 2–3 times with PBS, and fixed in 4% PFA for 15 min and washed again with precooled PBS 2–3 times. Then, the cells were permeabilized with 0.5% Triton X-100 for 10 min and washed 2–3 times with precooled PBS for 3–5 min each. The cells were stained with 0.5 μg/ml rhodamine-conjugated phalloidin in the dark for 40 min. After incubation, the cells were washed twice with precooled PBS for 3–5 min each. Finally, the nuclei were counterstained with 1 μg/ml DAPI in the dark for 3 min, washed twice with precooled PBS for 3–5 min and observed under fluorescence microscopy (Nikon 80i, Japan).

### 2.10 Acridine Orange Staining

Primary BMMs on glass chamber slides were induced with 10 ng/mL M-CSF and 10 ng/ml RANKL for 4 days and then treated with acacetin (0 and 20 μM) for another 24 h. The cells were washed 2–3 times with PBS and incubated with 5 μg/ml acridine orange for 15 min at 37°C. Then, the cells were washed with PBS and chased with fresh medium for 10 min, and stained cells were observed under fluorescence microscopy (Nikon 80i, Japan).

### 2.11 Preparation of Conditioned Media From Osteoclasts

Conditioned media from osteoclasts treated with acacetin (0, 5, 10, 20 μM) was prepared. BMMs (2 × 10^5^ cells/well) were seeded into 24-well plates, incubated with 10 ng/mL M-CSF and 10 ng/ml RANKL, and treated with various concentrations of acacetin (0, 5, 10, and 20 μM) for 4 days. At the end of induction, serum-containing conditioned medium from the mature osteoclasts was collected. Serum-free conditioned medium containing the same concentrations of M-CSF, RANKL, and acacetin was harvested after another day of culture. All the aliquoted conditioned media were centrifuged at 2,500 rpm for 10 min and stored at −80°C.

### 2.12 Tube Formation Assay

EPCs were seeded onto Matrigel-coated 24-well plates and incubated with acacetin or serum-containing conditioned medium from osteoclasts cultured with acacetin (0, 5, 10, and 20 μM). After 6 h, the cells were stained with calcein AM, and network formation was imaged using an inverted fluorescence microscope (Leica, Wetzlar, Germany). The total tube lengths and numbers of intersections in randomly chosen areas were quantified using ImageJ software.

### 2.13 Wound Healing Assay

EPCs were seeded into 6-well plates and grown to 90% confluence. An injury was created in the cell monolayer using a sterile 200 ml pipette, and unattached cells were removed by washing twice with PBS. Then, the cells were allowed to migrate into the empty space for 18 h in acacetin or serum-free conditioned medium from osteoclasts cultured with acacetin (0, 5, 10, and 20 μM). The cells were imaged during migration using an inverted optical microscope (Leica, Wetzlar, Germany). The width of the injury was measured using ImageJ software.

### 2.14 ELISA

BMMs (2 × 10^5^ cells/well) were seeded into 24-well plates, incubated with 10 ng/mL M-CSF and 10 ng/ml RANKL, and treated with various concentrations of acacetin (0, 5, 10, and 20 μM) for 4 days. The conditioned medium was subjected to PDGF-BB ELISA analysis using a mouse PDGF-BB ELISA kit according to the manufacturers’ instructions.

### 2.15 Animals

Eight-week-old C57BL/6 female mice were purchased from Beijing Vital River Laboratory (Beijing, China). Mice were randomly divided into three groups (*n* = 6 in each group), including the sham-operated (sham) group, the OVX group, and the OVX with acacetin (OVX + Acacetin) group. The sham group was treated with 0.5% CMC-Na with a sham operation, while both the OVX and OVX + Acacetin groups were ovariectomized, and orally administered 0.5% CMC-Na or 20 mg/kg/d acacetin for 8 weeks, respectively. The choice of dosage was based on previous studies (Ren et al., 2020; Wei et al., 2020). All animal experiments were performed in accordance with the Guiding Principles for the Care and Use of Laboratory Animals, and all experimental procedures were approved by the Institutional Experimental Animal Committee of Northwestern Polytechnical University (Xi’an, China).

### 2.16 Tissue Collection and Sample Preparation

Blood was collected from the hearts of mice before euthanasia after a 4-h fast. The collected blood was centrifuged at 1,500 g for 15 min, and the supernatant was collected and stored in a refrigerator at −80°C for later use. The femurs, tibiae and vertebrae were collected, and the attached muscles were removed. The left femurs were fixed in 4% PFA for 2 days and then embedded in paraffin after 4 weeks of decalcification with 10% EDTA. They were prepared for subsequent H&E staining, TRAP staining, immunohistochemical staining, and immunofluorescent staining. The right femurs and vertebrae were fixed in 4% PFA for micro-CT analysis. The left and right tibiae were collected and placed in a freezer at −80°C for real-time PCR analysis of acid phosphatase 5 (Acp5) and Rankl/osteoprotegerin (Opg) mRNA expression.

### 2.17 Serum Analysis

Serum was evaluated for AST, ALT, BUN, and CREA content using assay kits according to the manufacturer’s guidelines for each.

### 2.18 Micro-CT Analysis

The distal metaphysis of the femur and 5th lumbar vertebrae were fixed in 4% PFA and scanned using a Bruker SkyScan 1,276 Micro-CT device (Allentown, PA, United States). In brief, the scanning parameters were set as following, energy: 70 kV, 114 μA; angle of increment: 0.2°, exposure time: 810 ms/frame, scanning time: 14 min, and the scanning resolution were set as 10 μm. For femur, a region of interest (ROI) that start from the growth plate to 1.5 mm below the growth plate was selected for analysis (threshold = 117.4 mg HA/ccm) using CTvox software (Blue Scientific, Cambridge, United Kingdom), and three-dimensional (3D) reconstruct by NRecon Reconstruction software (Micro Photonics, Allentown, PA, United States). For vertebrae, the ROI of trabecular bone of 5th vertebral body was selected for analysis (threshold = 282.15 mg HA/ccm) and 3D reconstruction. The following parameters of trabecular bone and vertebrae were calculated using the direct three-dimensional measurement method: bone mineral density (BMD; g/cm^3^), bone surface area/bone volume (BS/BV; 1/mm), bone surface to tissue volume (BS/TV; 1/mm), bone volume to tissue volume (BV/TV; %), trabecular number (Tb.N; 1/mm), trabecular spacing (Tb.Sp; mm), and trabecular thickness (Tb.Th; mm).

### 2.19 Immunofluorescence, Immunohistochemistry, and Histomorphometry

Femurs of sham, OVX and OVX + Acacetin mice were embedded in paraffin and sectioned into 4-μm-thick tissue sections. For immunofluorescence analyses, sections were treated with 3% H_2_O_2_ for 25 min and then blocked with 3% BSA for 30 min. Then bone sections were incubated with individual primary antibodies against mouse CD31 (1:100), EMCN (1:100), PDGF-BB (1:100), and TRAP (1:100) overnight at 4°C. Then, the sections were washed and incubated with secondary antibodies conjugated with fluorescence in the dark for 1 h. Finally, the nuclei were counterstained with 1 μg/ml DAPI in the dark for 3 min and washed twice with precooled PBS for 3–5 min. CD31^hi^EMCN^hi^ vessels were quantitatively analysed as previously described (Xie et al., 2014). The number of positively stained cells in the field of distal femoral metaphysis was calculated and normalized to the number per square millimetre (N·mm^−2^) of bone marrow area in the trabecular bone. For immunohistochemistry analyses (Zhihao Chen et al., 2020), sections were processed for antigen retrieval for 15 min and blocked in 3% BSA for 30 min. The sections were then incubated overnight with primary antibody against CTSK (1:100) at 4°C, and incubated with HRP-conjugated IgG (1:400) for 50 min after three washes with PBS. A DAB Horseradish Peroxidase Colour Development Kit was used to detect immunoactivity, followed by counterstaining with haematoxylin. Sections were examined under a fluorescence microscope (Nikon, Japan). For bone histomorphometry, sections were stained with H&E. All sections were scanned using an Aperio AT2 Digital Whole Slide Scanner (Leica, Wetzlar, Germany).

### 2.20 Osteoblast Osteoclast Co-Culture

A transwell assay with polycarbonate membranes (0.4 μm pore size) (Corning Costar, MA, United States) was used for osteoblast-osteoclast co-culture. MC3T3-E1 cells (5 × 10^4^) were added to the upper compartment of the transwell system in α-MEM complete medium, and BMMs were added into lower chamber in α-MEM complete medium without RANKL or M-CSF. After 5 days co-culture, the cells in both upper and lower chamber were selected for further study.

### 2.21 Statistical analysis

The data are presented as the mean ± s.d. Analyses were performed using GraphPad Prism software. A Student’s *t-*test or ANOVA was performed to assess statistical significance of differences. Values were considered statistically significant at *p < 0.05 or **p < 0.01.

## 3 Results

### 3.1 The Cytotoxic Effect of Acacetin on BMMs

**FIGURE 1:**
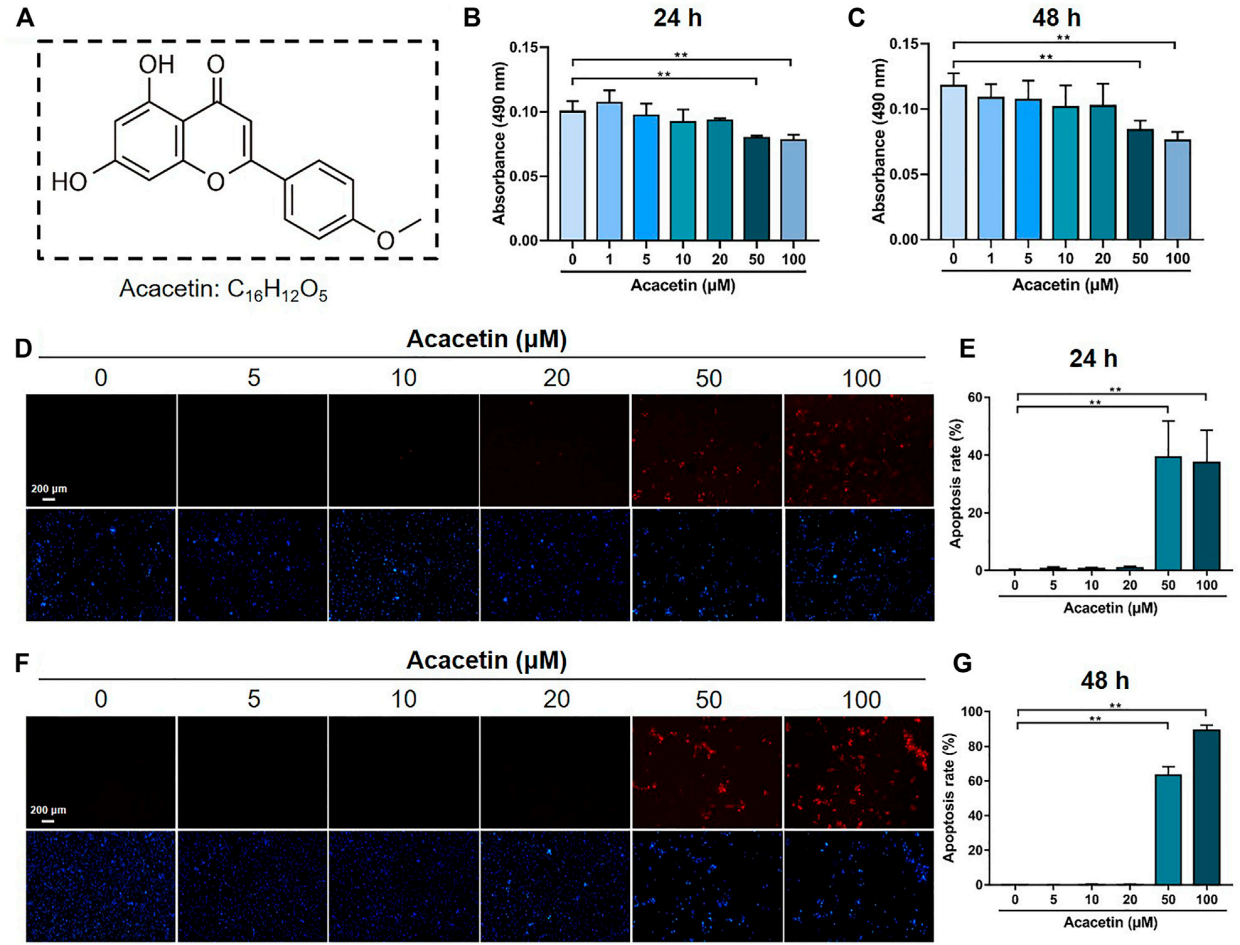
The cytotoxic effect of acacetin on BMMs. **(A)** Chemical structure of acacetin. **(B,C)** BMMs were treated with M-CSF and different concentrations of acacetin (0, 1, 5, 10, 20, 50, and 100 μM) for 24 h **(B)** and 48 h (C), and cell viability was measured by MTT assay (*n* = 6). **(D,F)** TUNEL staining of BMMs treated with M-CSF and different concentrations of acacetin (0, 5, 10, 20, 50, and 100 μM) for 24 h **(D)** and 48 h **(F)**. **(E,G)** Quantification of apoptotic cells in **(D)** and **(F)** (*n* = 4). The data are presented as the mean ± s.d.; A Student’s *t-*test or ANOVA was performed to assess statistical significance of differences; *p < 0.05 and **p < 0.01 versus control group.

To evaluate the concentration range in which acacetin is not toxic to BMMs, BMMs were treated with different concentrations of acacetin (Figure 1A), and cell viability was evaluated by the MTT assay. The results showed that acacetin did not exert a cytotoxic effect on BMMs at concentrations ranging from 0–20 μM after cultured for 24 h (Figure 1B) and 48 h (Figure 1C). Moreover, the TUNEL staining assay showed that acacetin at concentrations of 50 and 100 μM significantly increased the proportion of apoptotic cells, while 0–20 μM acacetin had no effect on cell apoptosis at either 24 h or 48 h (Figures 1D–G), consistent with the MTT results. Therefore, acacetin was used in the concentration range of 0–20 μM in subsequent study.

### 3.2 Acacetin Attenuates RANKL-Induced Osteoclastogenesis and NFATc1 and NF-κB Expression

**FIGURE 2:**
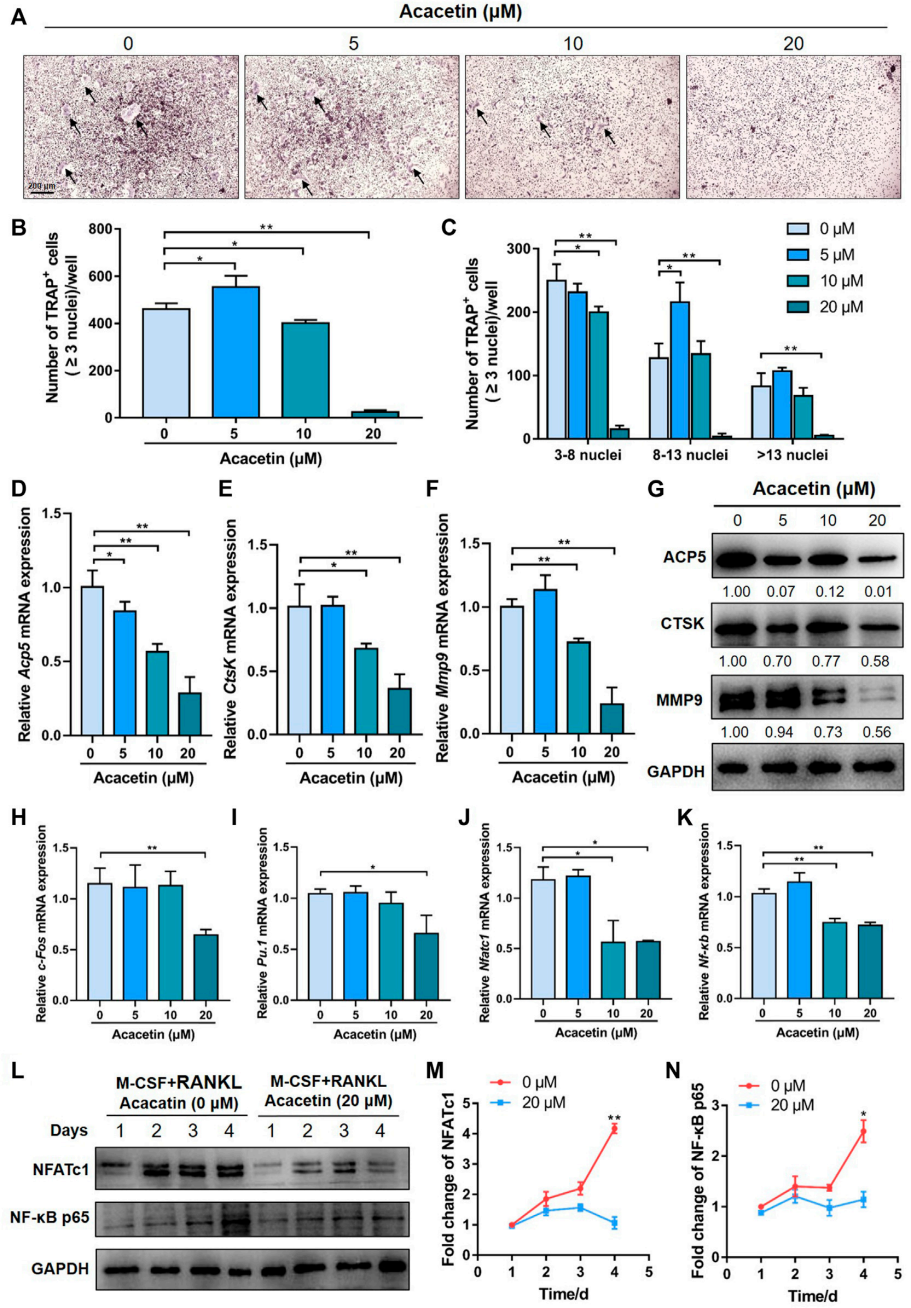
Acacetin attenuates RANKL-induced osteoclastogenesis. **(A)** Representative images of TRAP staining with the indicated concentrations of acacetin (0, 5, 10, and 20 μM) for 4 days. (Scale bar = 200 μm). **(B,C)** Quantification of multinucleated cells (≥3 nuclei) is shown in **(A)** (*n* = 3). **(D–F)** Real-time PCR analysis of *Acp5*, *Ctsk*, and *Mmp9* expression in osteoclasts treated with different concentrations of acacetin (0, 5, 10, and 20 μM) (n = 4) (G) Western blotting analysis of Acp5, Ctsk, and Mmp9 expression in osteoclasts treated with acacetin (0, 5, 10, and 20 μM). **(H–K)** Real-time PCR analysis of *c-fos*, *Pu.1*, *Nfatc1*, and *Nf-κb* expression in osteoclasts treated with 20 μM acacetin (*n* = 3). **(L)** Western blotting analysis of NFATc1 and NF-κB expression during osteoclastogenesis treated with 20 μM acacetin. **(M,N)** Quantification of NFATc1 and NF-κB levels versus GAPDH levels (*n* = 3). The data are presented as the mean ± s.d.; A Student’s *t-*test or ANOVA was performed to assess statistical significance of differences; *p < 0.05 and **p < 0.01 versus control group.

To investigate the potential role of acacetin in RANKL-induced osteoclast differentiation, freshly harvested BMMs were cultured with 10 ng/mL M-CSF and 10 ng/ml RANKL with or without different concentrations of acacetin. After 4 days of induction, TRAP staining revealed that acacetin inhibited the size and number of osteoclasts, and the formation of multinucleated osteoclasts (≥3 nuclei), and the effect was most obvious at 20 μM (Figures 2A–C). Interestingly, real-time PCR results showed that the osteoclastic marker genes *Acp5*, *CtsK*, and *Mmp9* were significantly downregulated in the 10 and 20 μM acacetin-treated groups (Figures 2D–F). Additionally, western blotting analysis indicated that 20 μM acacetin had the most obvious inhibitory effect on the protein expression of CTSK, ACP5, and MMP9 (Figure 2G). Taken together, these results demonstrate that 20 μM acacetin inhibits RANKL-induced osteoclast differentiation.

To investigate the signalling pathways by which acacetin inhibits osteoclast formation, M-CSF- and RANKL- induced osteoclasts were examined for expression of osteoclast-related transcription factors in response to different concentrations of acacetin (0, 5, 10, and 20 μM). Real-time PCR results showed that expression of *c-Fos* and *Pu.1* were reduced by 20 μM acacetin after 4 days of induction (Figures 2H, I), while the expression of *Nfatc1* and *Nf-κb* was significantly reduced by acacetin at both 10 and 20 μM (Figures 2J, K). Furthermore, the western blotting results was consistent, and the protein expression of NFATc1 and NF-κB p65 was significantly reduced by 20 μM acacetin during the osteoclastogenesis process (Figures 2L–N). These data indicate that acacetin abrogates RANKL-induced NFATc1 and NF-κB expression.

### 3.3 Acacetin Attenuates Bone Resorption and Acidification of Osteoclasts

**FIGURE 3:**
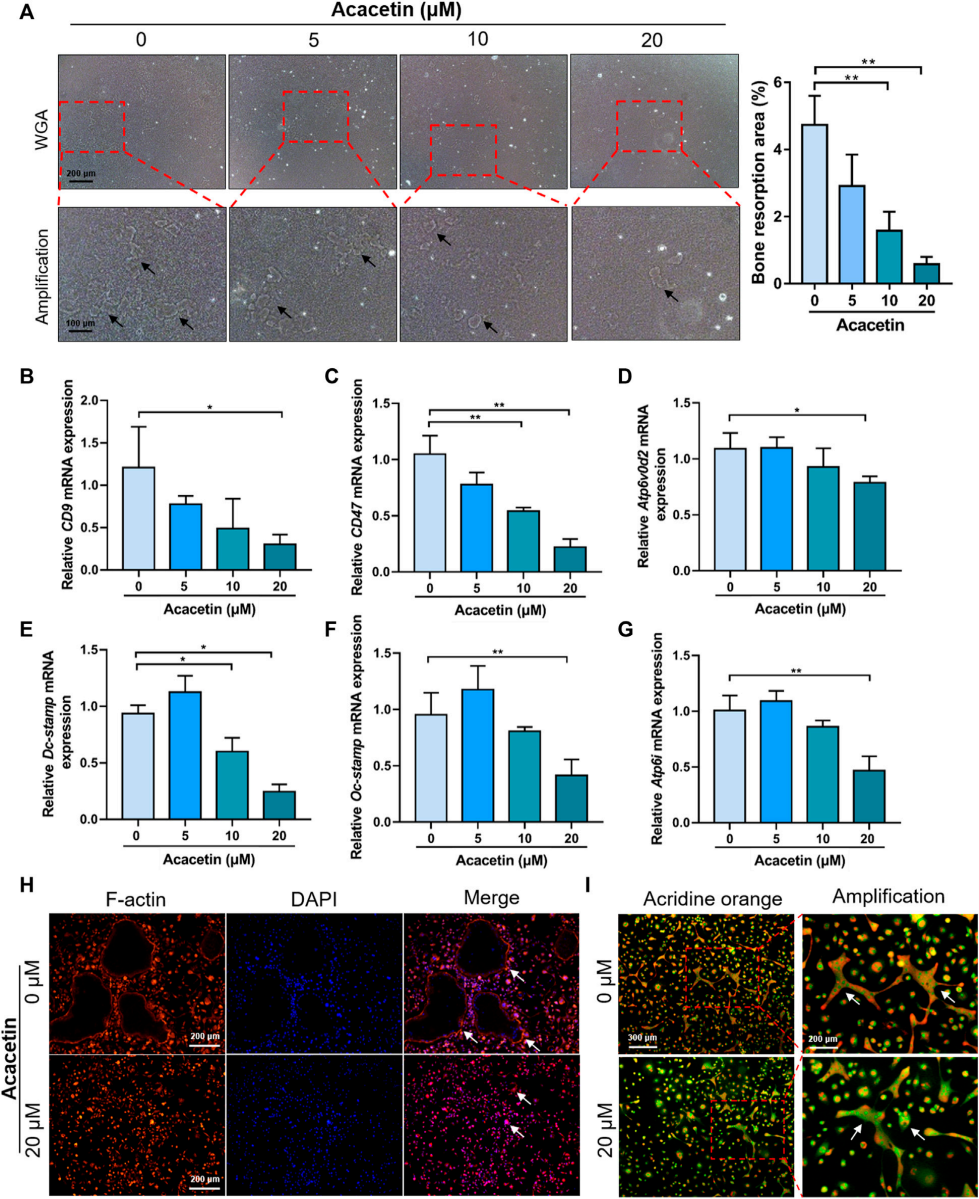
Acacetin attenuates bone resorption and acidification of osteoclasts**. (A)** Representative images and quantitative analyses of bone resorption area of osteoclasts on osteo assay surface treated with acacetin (0, 5, 10, and 20 μM). (Upper: scale bar = 200 μm, lower: scale bar = 100 μm). **(B–G)** Real-time PCR analysis of *CD9*, *CD47*, *Atp6v0d2*, *Dc-stamp*, *Oc-stamp*, and *Atp6i* expression in osteoclasts treated with different concentrations of acacetin (0, 5, 10, and 20 μM) (*n* = 3). **(H)** Representative images of actin ring staining of osteoclasts treated with 20 μM acacetin. (Scale bar = 200 μm). **(I)** Representative images of acridine orange staining of osteoclasts treated with 20 μM acacetin. (Upper: scale bar = 300 μm, lower: scale bar = 200 μm). The data are presented as the mean ± s.d.; A Student’s *t-*test or ANOVA was performed to assess statistical significance of differences; A Student’s *t-*test or ANOVA was performed to assess statistical significance of differences; *p < 0.05 and **p < 0.01 versus control group.

To further study the role of acacetin in osteoclast function, we performed a bone resorption assay. Equal numbers of BMMs were cultured on the osteo assay surface and treated with 10 ng/mL M-CSF, 10 ng/ml RANKL for 5 days, and treated with acacetin at concentrations of 0, 5, 10, and 20 μM for another 2 days. Bone resorption assays indicate that the total resorption area was significantly reduced in the acacetin treatment groups (10 and 20 μM) (Figure 3A).

The fusion of pre-osteoclast is the key step of osteoclast function. To determine the effect of acacetin on osteoclast fusion, osteoclast fusion genes were further examined by real-time PCR. The results showed that expression of *CD9*, *CD47*, *Atp6v0d2*, *Dc-stamp*, and *Oc-stamp* was reduced in response to 20 μM acacetin treatment after 4 days of induction (Figures 3B–F). Moreover, small F-actin belts and few nuclei were observed in osteoclasts after 20 μM acacetin treatment (Figure 3H). Additionally, the expression of *Atp6i*, which determines extracellular acidification, was downregulated in acacetin-treated osteoclasts (Figure 3G). These results are consistent with those of acridine orange staining, that is, the level of extracellular acidification was lower after 20 μM acacetin treatment (Figure 3I). Taken together, these data indicate that 20 μM acacetin inhibits osteoclast acidification and bone resorption.

### 3.4 Acacetin Attenuates RANKL-Induced Osteoclastogenesis Through Akt/GSK3β and NF-κB Signalling

**FIGURE 4:**
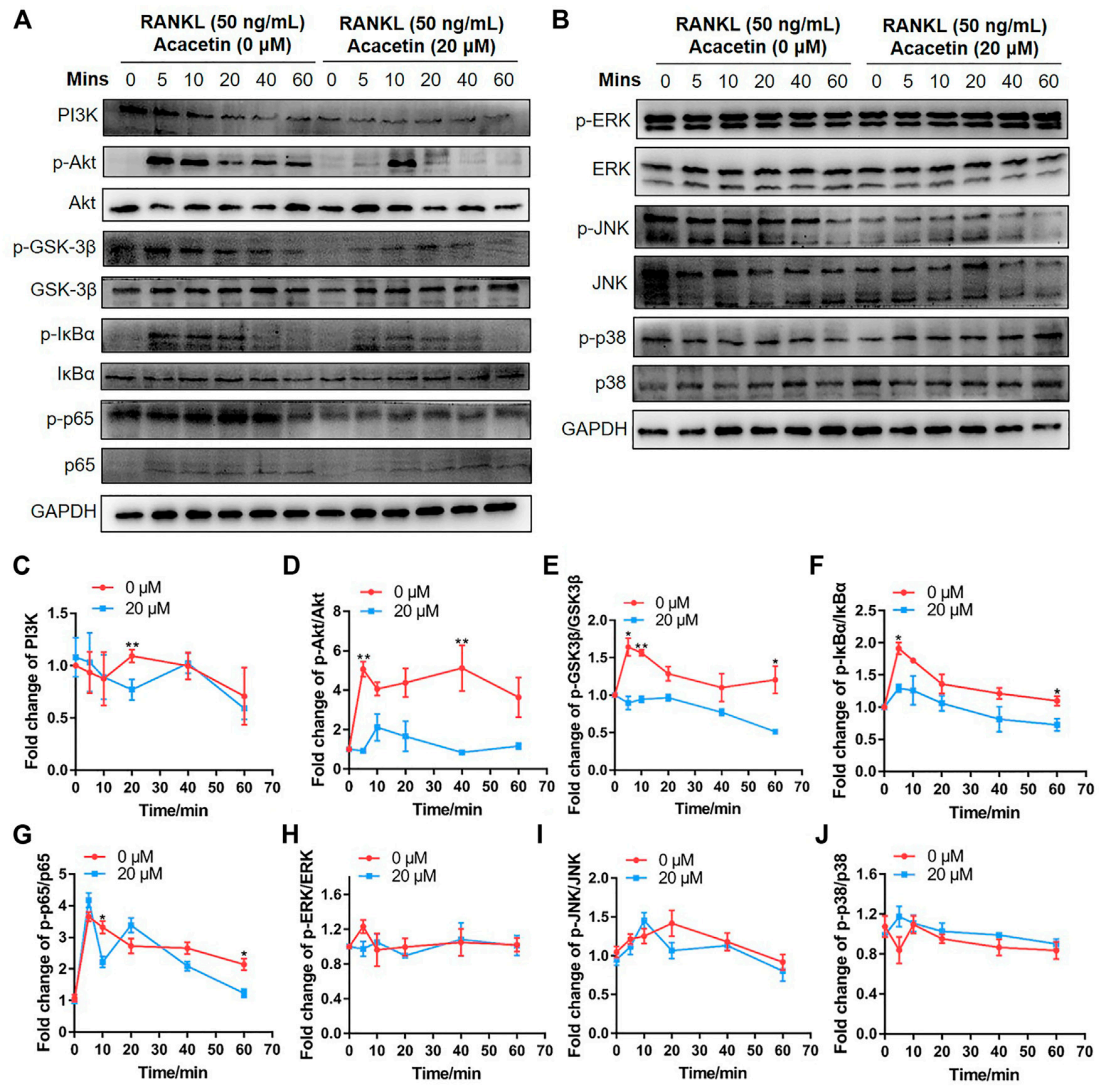
Acacetin attenuates RANKL-induced osteoclastogenesis through Akt/GSK3β and NF-κB signalling. **(A,B)** BMMs were induced with M-CSF and RANKL for 3 days, and starved for 5 h and stimulated with 50 ng/ml RANKL for the indicated times. Western blotting analysis with specific antibodies was performed as indicated. **(C–J)** Quantification of PI3K level versus GAPDH level, phospho-Akt level versus total Akt level, phospho-GSK3β level versus total GSK3β level, phospho-IκBα level versus total IκBα level, phospho-p65 level versus total p65 level, phospho-ERK level versus total ERK level, phospho-JNK level versus total JNK level, and phospho-p38 level versus total p38 level (*n* = 3). The data are presented as the mean ± s.d.; A Student’s *t-*test was performed to assess statistical significance of differences; *p < 0.05 and **p < 0.01 versus control group.

To further explore the molecular mechanism by which acacetin inhibits RANKL-induced osteoclast formation, BMMs were induced with 10 ng/mL M-CSF and 10 ng/ml RANKL for 3 days. Then, the cells were starved for 5 h and stimulated with 50 ng/ml RANKL or RANKL + acacetin for 5–60 min. Western blotting results demonstrated that phosphorylation of Akt, GSK3β, IκBα, and p65, which was increased by RANKL, was markedly attenuated by acacetin treatment (Figures 4A, C–G). However, RANKL-induced early signalling pathways, such as activation of ERK, JNK, and p38, were not altered by acacetin treatment (Figures 4B, H–J). These data suggest that acacetin attenuates RANKL-induced osteoclastogenesis by inhibiting the Akt/GSK3β and NF-κB signalling pathways.

### 3.5 Acacetin Promotes Preosteoclast-Induced Angiogenesis

**FIGURE 5:**
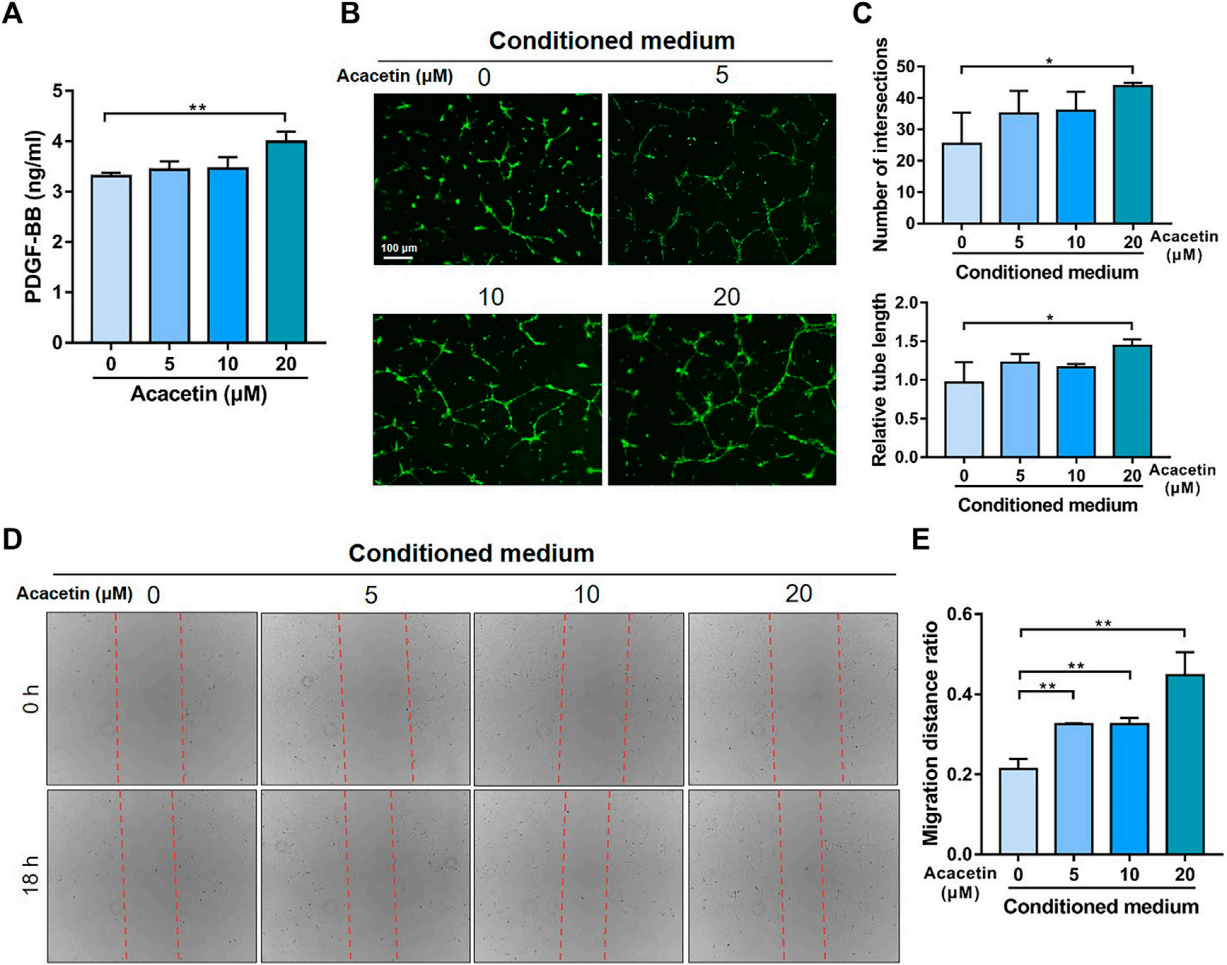
Acacetin promotes preosteoclast induced angiogenesis. **(A)** ELISA analysis of PDGF-BB levels in serum-containing conditioned medium from osteoclasts treated with acacetin (0, 5, 10, and 20 μM) (*n* = 3). **(B)** Representative Matrigel tube formation assay images with cultures of EPCs using conditioned medium from acacetin-treated osteoclast as indicated. (Scale bar = 100 μm). **(C)** Quantification of tube length and number of intersections in **(B)** using ImageJ (*n* = 3). **(D)** The mobility of EPCs in conditioned medium from acacetin-treated osteoclast as indicated was assessed by wound healing assays. **(E)** Quantification of migration distance in **(D)** using ImageJ (*n* = 3). The data are presented as the mean ± s.d.; A Student’s *t-*test or ANOVA was performed to assess statistical significance of differences; *p < 0.05 and **p < 0.01 versus control group.

To validate the effects of acacetin on preosteoclast-induced angiogenesis, the concentration of PDGF-BB in serum-containing conditioned medium from acacetin (0, 5, 10, and 20 μM) treated osteoclasts was measured. As evidenced by ELISA, the production of PDGF-BB in conditioned medium was increased by acacetin treatment at a concentration of 20 μM (Figure 5A). Furthermore, EPCs were cultured on Matrigel, incubated with serum-containing conditioned medium, and allowed to form capillary-like tubes. The results showed that acacetin promoted the angiogenesis process of EPCs with enhanced tube length and a higher number of intersections (Figures 5B, C). Additionally, wound healing assays revealed that serum-containing conditioned medium from acacetin treated osteoclasts promotes the migration of EPCs in a concentration-dependent manner (Figures 5D, E). To exclude the direct effect of acacetin on EPCs, the acacetin (0, 5, 10, and 20 μM) treated EPCs was measured. It was showed that acacetin inhibited both the angiogenesis and migration of EPCs (Supplementary Figure S1). Taken together, these findings indicate that acacetin promotes preosteoclast-induced angiogenesis.

### 3.6 Intragastric Administration of Acacetin Prevents Bone Loss Induced by OVX

**FIGURE 6:**
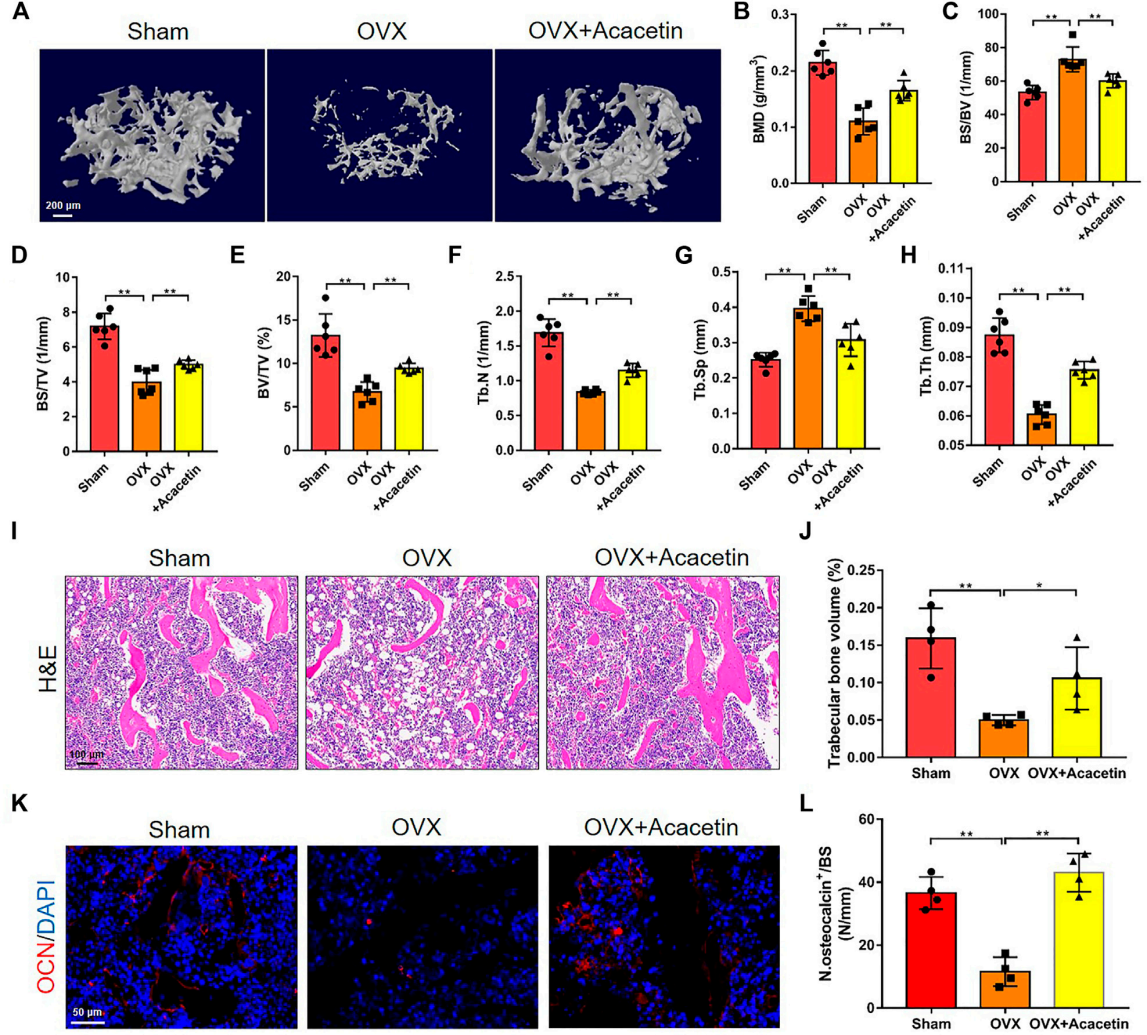
Intragastric administration of acacetin prevents bone loss induced by OVX. **(A)** Representative 3D reconstruction micro-CT images of femur trabecular bone in the different groups. (Scale bar = 200 μm). **(B–H)** Quantitative analyses of femur trabecular bone-related parameters, including BMD, BS/BV, BS/TV, BV/TV, Tb.N, Tb.Sp, and Tb.Th (*n* = 6). **(I)** Representative H&E staining images of distal femurs in sham group, OVX group, and OVX group treated with 20 mg/kg acacetin. (Scale bar = 100 μm). **(J)** Quantitative analyses of trabecular bone volume in **(I)** (*n* = 4). **(K)** Representative immunostaining of OCN on the trabecular bone surface. (Scale bar = 50 μm). **(L)** Quantitative analyses of OCN^+^ cell numbers on the trabeculae in **(K)** (*n* = 4). The ROI of microCT, H&E, and OCN is in the region that start from the growth plate to 1.5 mm below the growth plate. The data are presented as the mean ± s.d.; A Student’s *t-*test or ANOVA was performed to assess statistical significance of differences; *p < 0.05 and **p < 0.01 versus control group.

To evaluate the effects of acacetin on osteolytic disease, OVX mice were generated and intragastrically administered acacetin (20 mg/kg/d) or vehicle (CMC-Na) for 2 months. Serum analysis revealed that acacetin did not exert liver or kidney toxicity in mice because there was no significant change in AST, ALT, BUN, or CREA content (Supplementary Figures S2A–D). Micro-CT analysis revealed that the trabecular bone mass of OVX mice was markedly lower in the distal metaphysis of the femur than in the sham group, but the acacetin alleviated bone loss in OVX mice (Figure 6A). Quantitative analyses of BMD, BS/BV, BS/TV, BV/TV, Tb.N, Tb.Sp, and Tb.Th confirmed the preventive effect of acacetin on OVX-induced bone loss (Figures 6B–H). Moreover, micro-CT and quantitative analyses of vertebrae also demonstrated that acacetin inhibited bone loss in OVX mice (Supplementary Figures S2E–L). H&E staining showed that acacetin induced bone area and prevented fat cell accumulation in the bone marrow of OVX mice (Figures 6I, J). Additionally, immunofluorescence staining of osteocalcin (OCN) showed an increased number of osteoblasts on the surface of trabecular bone in acacetin-treated mice (Figures 6K, L). To determine whether acacetin treatment regulates osteoclasts through osteoblasts, BMMs were co-cultured with osteoblasts treated with acacetin (0, 5, 10, and 20 μM) without RANKL or M-CSF. The osteoblast differentiation marker genes including *Runx2*, *Alp* and *Ocn* were upregulated by acacetin, while the expression of *Rankl*/*Opg* was not changed (Supplementary Figure S3A). Furthermore, the osteoclastic marker genes *Acp5*, *CtsK*, and *Mmp9* were not changed in osteoclast co-cultured with osteoblasts treated with acacetin (Supplementary Figure S3). These results indicate that the inhibition of osteoclast differentiation by acacetin is direct. Altogether, these results indicate that acacetin effectively protects against OVX-induced osteoporosis *in vivo*.

### 3.7 Acacetin Represses Osteoclasts and Stimulates Type H Vessel Formation in OVX Mice

**FIGURE 7:**
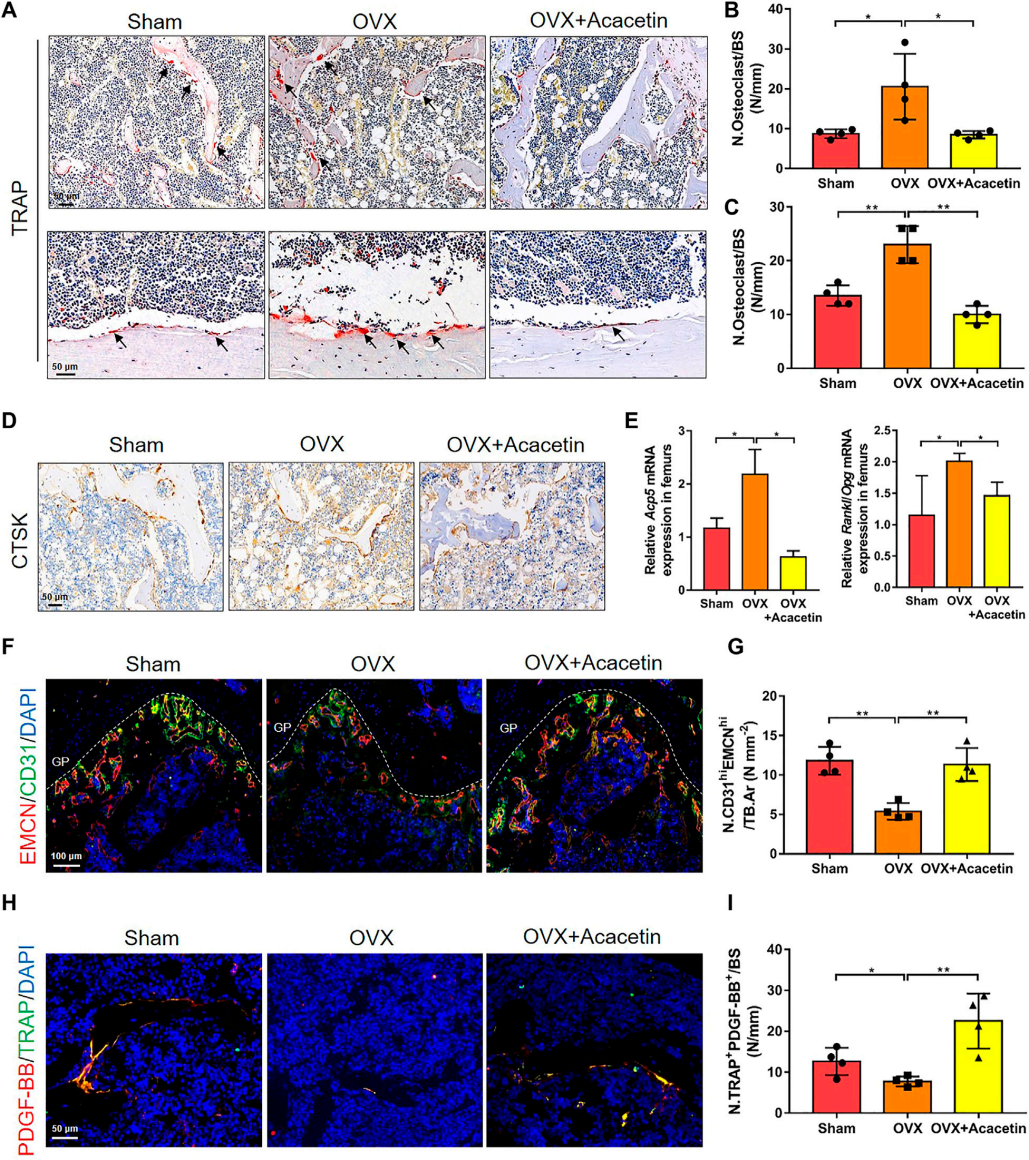
Acacetin represses osteoclasts and stimulates type H vessel formation in OVX mice. **(A)** Representative TRAP^+^ cells on the trabecular bones of distal femurs (upper) and cortical bone of diaphyseal femurs (below). (Scale bar = 50 μm). **(B,C)** Quantification of TRAP staining in trabecular bones of distal femurs **(B)** and cortical bone of diaphyseal femurs **(C)** (*n* = 4). **(D)** Representative immunohistochemistry of CTSK^+^ cells on the trabecular bone surface. (Scale bar = 100 μm). **(E)** Real-time PCR analysis of *Acp5* and *Rankl/Opg* expression in bone tissues (*n* = 3). **(F)** Representative images of immunostaining of endomucin (EMCN) (red) and CD31 (green) on trabecular bone (Scale bar = 100 μm). **(G)** Quantification of CD31^hi^EMCN^hi^ (yellow) cells in bone marrow in **(F)** (*n* = 4). **(H)** Representative images of immunostaining of PDGF-BB (red) and TRAP (green) on the trabecular bone (Scale bar = 50 μm). **(I)** Quantification of PDGF-BB^+^ TRAP^+^ (yellow) cells in bone marrow in **(H)** (*n* = 4). The ROI of TRAP, Ctsk, and PDGF-BB/TRAP staining was in the region that start from the growth plate to 1.5 mm below the growth plate. The ROI of EMCN/CD31 staining is in the region of growth plate. The data are presented as the mean ± s.d.; A Student’s *t-*test or ANOVA was performed to assess statistical significance of differences; *p < 0.05 and **p < 0.01 versus control group.

To investigate osteoclast development *in vivo*, we analysed sections of the femora by TRAP staining. The results showed that TRAP^+^ osteoclasts on the trabecular bone of distal femurs and cortical bone were increased, while acacetin reduced the number of osteoclasts in OVX mice (Figures 7A–C). Immunohistochemical staining for CTSK revealed that osteoclastogenic activity was promoted in OVX mice but significantly inhibited when OVX mice were treated with acacetin (Figure 7D). Moreover, the expression of *Acp5* and *Rankl*/*Opg* in the femurs of OVX mice was upregulated in response to acacetin treatment (Figure 7E).

An increase in the number of preosteoclasts induces the formation of CD31^hi^Emcn^hi^ vessels (Xie et al., 2014). Immunofluorescence staining showed that acacetin promoted the formation of CD31^hi^EMCN^hi^ vessels adjacent to the growth plate (GP) of OVX mice, which was similar to the sham-operated mice (Figures 7F, G). Preosteoclasts couple angiogenesis and bone formation by secreting PDGF-BB (Xie et al., 2014). Therefore, PDGF-BB and TRAP immunofluorescence double staining was performed to further evaluate the distribution of preosteoclasts expressing PDGF-BB in the femur. The results revealed that OVX mice had a significantly lower proportion of TRAP^+^ cells that were positive for PDGF-BB than sham-operated mice, while acacetin rescued the proportion of PDGF-BB^+^/TRAP^+^ cells in OVX mice (Figures 7H, I). Collectively, these results suggest that acacetin reduces the number of multinuclear osteoclasts and increases the number of PDGF-BB^+^/TRAP^+^ preosteoclasts that stimulate angiogenesis and bone formation in OVX mice.

## 4 Discussion

Osteoporosis is a common bone disease characterized by excessive bone resorption mediated by osteoclasts and resulting in bone loss (Qiang Xu et al., 2021). It is estimated that more than 200 million people currently suffer from osteoporosis (Tatangelo et al., 2019). As the population continues to age and live longer, the number of people affected will increase significantly. Therefore, osteoporosis is considered a serious public health problem worldwide (Akbar et al., 2017).

To prevent the development of osteoporosis, a great deal of work has been done to identify effective treatments. Overactivation of osteoclasts plays a major role in bone destruction, therefore, osteoclast differentiation is considered as a major therapeutic target for developing new drugs (Ono and Nakashima, 2018). Clinically, antiresorptive agents, such as alendronate, zoledronate, and denosumab, are widely used for the treatment of osteoporosis (Leder et al., 2020). Unfortunately, these drugs can cause a number of side effects, including stroke, gastrointestinal discomfort, hypokalaemia, and osteonecrosis of the jaw (Chen et al., 2019; Delong Chen et al., 2020). Considering these shortcomings, it is imperative to find effective drugs to treat unbalanced bone remodelling caused by excessive osteoclast activity (Huang et al., 2015; Wantao Li et al., 2020). Flavonoids have antioxidant, anti-inflammatory, differentiation, and apoptotic properties (Bellavia et al., 2021), which make them important in maintaining bone health (Hardcastle et al., 2011; Zhang et al., 2014). Recently, studies have shown that a large number of compounds, such as cladrin, icariin, and petunidin have inhibitory effects on osteoclast function and represent potential therapies for bone resorption diseases (Bellavia et al., 2021). Acacetin has been reported to promote osteoblastic differentiation and mineralization (Li et al., 2016) and inhibit osteoclastic differentiation through regulating CD44 and integrins *in vitro* (Kim et al., 2020). However, the effect and mechanism of acacetin on the formation of osteoclasts and type H vessels in OVX-induced bone loss have not yet been elucidated. In this study, we found that acacetin inhibits macrophages from differentiating into multinucleated osteoclasts. Relative expression of osteoclast marker genes, including *Acp5*, *Ctsk*, and *Mmp9*, also demonstrated the inhibitory effects of acacetin on osteoclastogenesis. Next, we tested the role of acacetin in osteoclast function. The bone resorption assay, actin-ring staining, and acridine orange staining confirmed that acacetin inhibited the bone resorption and extracellular acidification of osteoclasts. These results indicate that acacetin suppresses RANKL-induced osteoclast formation and function *in vitro*.

The binding of RANKL to RANK during osteoclast differentiation activates key downstream signalling pathways, such as Akt and NF-κB (Moon et al., 2012; Chen et al., 2018; Ilchovska and Barrow, 2021). Previous studies have shown that Akt induces osteoclast differentiation by inhibiting the GSK3β signalling cascade (Wu et al., 2017). Acacetin has been reported to bind with the p110α subunit of PI3K (Jung et al., 2014). Here, we found that acacetin limits the phosphorylation of Akt and GSK3β, which are downstreams of PI3K, in response to RANKL stimulation and that acacetin regulates expression of the transcription factor NFATc1 expression during the late stage of osteoclast differentiation. Moreover, acacetin inhibited NF-κB signalling in inflammation-associated tumorigenesis and osteoarthritis (Pan et al., 2006; Jian Chen et al., 2020). In our study, acacetin inhibited p65 and IκBα phosphorylation at 10 and 60 min of RANKL stimulation, suggesting that acacetin responds to RANKL signaling during the early and late stages of osteoclast formation. c-Fos and pu.1 can be activated by RANKL and induce gene expression with NFATc1 during osteoclast formation (Takayanagi et al., 2002). c-Fos deficient mice exhibit reduced NFATc1 expression and an osteoporotic phenotype (Grigoriadis et al., 1994). Furthermore, the development of osteoclasts and macrophages is inhibited in the pu.1-deficient mice, inducing an osteosclerosis phenotype (Tondravi et al., 1997). Here, we found that the mRNA expression of c-Fos and pu.1 was significantly reduced in RANKL-stimulated BMMs in response to acacetin treatment. This expression trend was consistent with the marker genes for osteoclast differentiation.

Type H vessels play an important role in maintaining normal bone structure (Kusumbe et al., 2014; Ramasamy et al., 2016). Previous studies have shown that preosteoclasts significantly affect bone formation, and the destruction of type H blood vessels is one of the primary causes of reduced bone formation (Xie et al., 2014; Yang et al., 2018; Gao et al., 2019). The number of type H endothelial cells and bone progenitor cells was significantly reduced in the bone of osteoporosis (Wang et al., 2017; Zhu et al., 2019). Therefore, type H vessels play an important role in bone formation and osteoporosis. It has been reported that acacetin could inhibit angiogenesis *in vitro* and *in vivo* (Bhat et al., 2013). However, the influence of acacetin on the formation of type H vessels through osteoclasts is unclear. In this study, we found that conditioned medium from osteoclasts incubated with RANKL and acacetin enhanced the migratory ability and tube structure formation of EPCs. These results are consistent with previous reports that reduced multinucleated osteoclasts and enhanced preosteoclasts contributed to type H vessel formation.

Finally, a preclinical study using an OVX mouse model of osteoporosis confirmed the role of acacetin in reducing bone loss. The micro-CT results showed that treatment with acacetin restored OVX-induced damage to the trabecular bone architecture of the femur and vertebrae via increases in BMD, BS/TV, BV/TV, Tb.N, and Tb.Th, and a decrease in the BS/BV and Tb. Sp parameters. Moreover, acacetin increased the number of osteoprogenitor cells in the femurs. Our results demonstrated that OVX-induced increases in the number of osteoclasts and enhanced expression of *Acp5* and *Rankl*/*Opg* in bone were significantly reduced by acacetin treatment. Type H vessels play an important role in maintaining normal bone structure (Kusumbe et al., 2014; Ramasamy et al., 2016). Previous studies have shown that preosteoclasts significantly affect bone formation through PDGF-BB, and the destruction of type H blood vessels is one of the primary causes of reduced bone formation (Xie et al., 2014; Yang et al., 2018; Gao et al., 2019). The number of type H endothelial cells and bone progenitor cells was significantly reduced in the bone of osteoporosis (Wang et al., 2017; Zhu et al., 2019). Therefore, type H vessels play an important role in bone formation and osteoporosis. However, the influence of acacetin on the formation of type H vessels is unclear. In this study, we found that type H vessels and TRAP^+^PDGF-BB^+^ cells were induced by acacetin in OVX mice. Taken together, these observations demonstrated that acacetin exerts a protective effect on bone loss *in vivo*.

## 5 Conclusion

In summary, we demonstrated that acacetin prevents OVX-induced bone loss by regulating bone resorption and type H vessel formation. Notably, our results reveal that acacetin treatment inhibits osteoclastogenesis and bone resorption through suppression of the Akt/GSK3β and NF-κB signalling pathways. Therefore, this study provides potential application prospects for the pharmacological countermeasures of acacetin in osteoporosis treatment.

## Data Availability

The original contributions presented in the study are included in the article/Supplementary Material, further inquiries can be directed to the corresponding authors.
