# Supplementary material for: Acacetin Prevents Bone Loss by Disrupting Osteoclast Formation and Promoting Type H Vessel Formation in Ovariectomy-Induced Osteoporosis
Source: Front Cell Dev Biol. 2022 Apr 19;10:796227. doi: 10.3389/fcell.2022.796227 (PMC9062130; doi:10.3389/fcell.2022.796227)
Supplement: Supplementary file 1 [file Image1.pdf]

Supplementary Material

1 Supplementary Figures

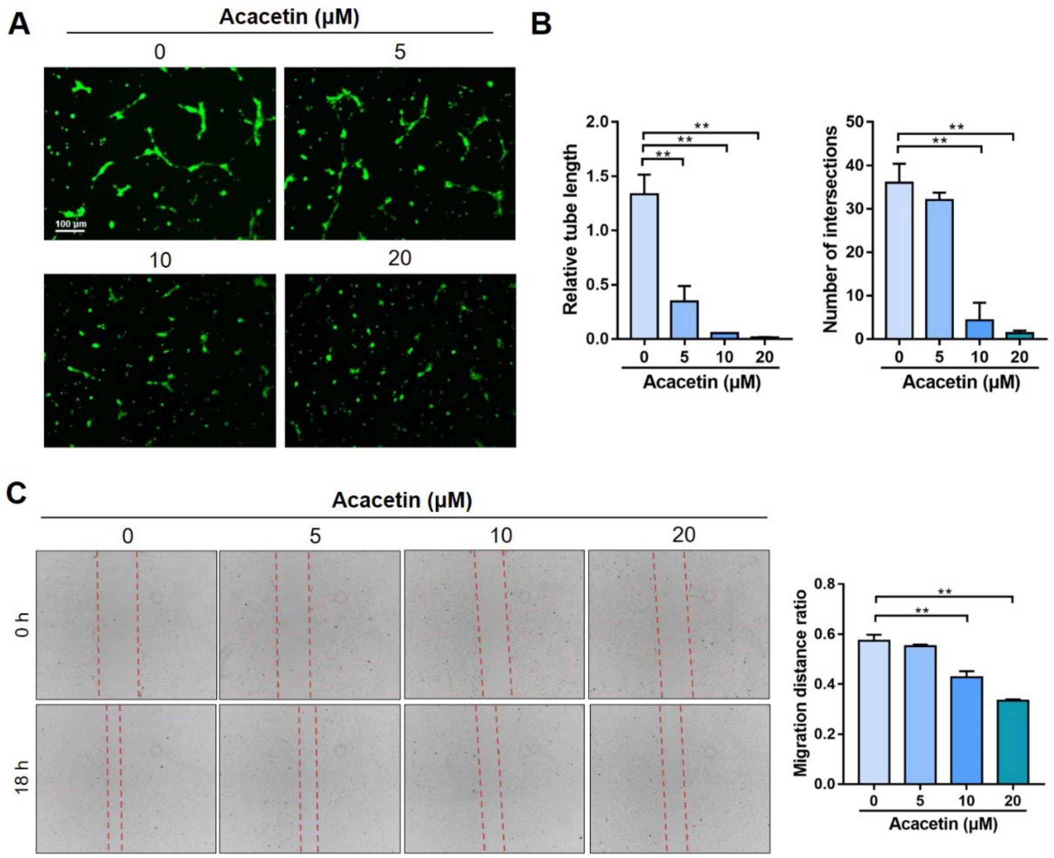

Supplementary Figure 1. Acacetin inhibits the angiogenesis and migration of EPCs directly. (A) Representative Matrigel tube formation assay images with cultures of EPCs with acacetin directly as indicated. (Scale bar=100 μm). (B) Quantification of tube length and number of intersections in (A) using ImageJ (n=3). (C) The mobility of EPCs with acacetin directly as indicated was assessed by wound healing assays, and the migration distance was quantified using ImageJ (n=3). The data are presented as the mean ± s.d.; A Student's *t*-test or ANOVA was performed to assess statistical significance of differences; *$P < 0.05$ and **$P < 0.01$ versus control group.

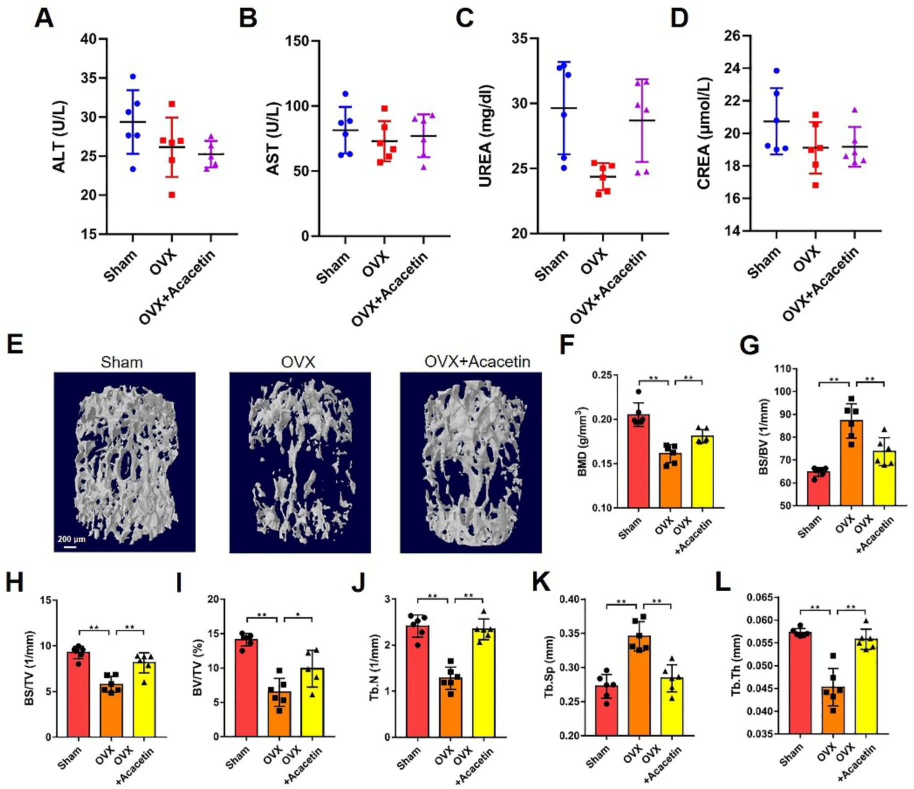

Supplementary Figure 2. Acacetin prevents bone loss induced by OVX. (A-D) Serum analysis of AST, ALT, BUN, and CREA content. (E) Representative 3D reconstruction micro-CT images of vertebral bone in the different groups. (Scale bar=200 μm). (F-L) Quantitative analyses of vertebral bone-related parameters, including BMD, BS/BV, BS/TV, BV/TV, Tb.N, Tb.Sp, and Tb.Th. The data are presented as the mean ± s.d.; A Student's *t*-test or ANOVA was performed to assess statistical significance of differences; *$P < 0.05$ and **$P < 0.01$ versus control group.

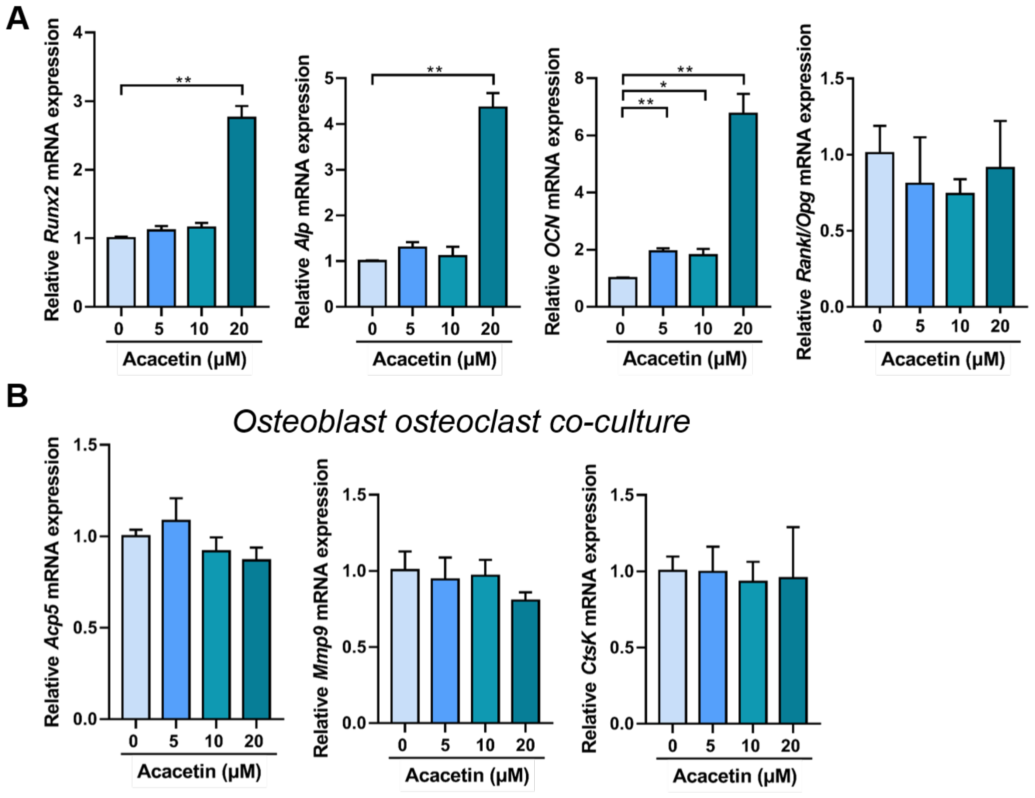

Supplementary Figure 3. Acacetin promoted differentiation of osteoblasts and could not regulate osteoclasts through osteoblast. (A) Real-time PCR analysis of *Runx2*, *Alp*, *Ocn* and *Rankl*/*Opg* expression in osteoblasts treated with different concentrations of acacetin (0, 5, 10, and 20 μM) (n=4). (B) Real-time PCR analysis of *Acp5, Mmp9* and *Ctsk* expression in osteoclasts co-cultured with osteoblasts treated with different concentrations of acacetin (0, 5, 10, and 20 μM) (n=4). The data are presented as the mean ± s.d.; A Student's *t*-test or ANOVA was performed to assess statistical significance of differences; *$P < 0.05$ and **$P < 0.01$ versus control group.
